# Answering the Call for Diversity and Racial Equity: The National Association for Geriatric Education

**DOI:** 10.1093/geroni/igab046.340

**Published:** 2021-12-17

**Authors:** Katherine Bennett, Rosellen Rosich, Linda Edelman, Barbara Gordon, Anna Goroncy, Jennifer Severance

**Affiliations:** 1 University of Washington, Seattle, Washington, United States; 2 University of Alaska Anchorage, Anchorage, Alaska, United States; 3 University of Utah College of Nursing, Salt Lake City, Utah, United States; 4 University of Louisville, Louisville, Kentucky, United States; 5 University of Cincinnati Department of Family and Community Medicine , Cincinnati, Ohio, United States; 6 University of North Texas Health Science Center - Ft. Worth, TX, Fort Worth, Texas, United States

## Abstract

The National Association for Geriatric Education (NAGE) is a non-profit organization representing geriatric and gerontology programs, including Health Services and Resource Administration funded Geriatric Workforce Enhancement Programs (GWEPs), and Geriatric Academic Career Awardees (GACAs). NAGE responded to the renewed call to address systemic racism and racial inequities by forming a Diversity and Racial Equity Workgroup. The Workgroup explored ways to disseminate educational resources, support members to address racial inequities among older adults, promote increased diversity of the geriatrics/gerontology workforce, and support public policy initiatives that address racism and health disparities. Initial outputs include creating a Diversity and Racial Equity resource page, identifying liaisons to the Workgroup from each NAGE Committee to ensure impact across the organization, and organizing collaborations across GWEPs and GACAs to share successful initiatives. Future plans include education and advocacy with members and collaborating organizations to address systemic racism and racial health inequities impacting older adults.

